# ICTV Virus Taxonomy Profile: Turriviridae 2024

**DOI:** 10.1099/jgv.0.002000

**Published:** 2024-07-03

**Authors:** Sydnie K. Chase, Jamie C. Snyder

**Affiliations:** 1 Department of Biological Sciences, Cal Poly Pomona, Pomona, CA 91768, USA

**Keywords:** archaeal viruses, ICTV report, STIV, *Sulfolobus*, taxonomy, *Turriviridae *

## Abstract

The family *Turriviridae* includes viruses with a dsDNA genome of 16–17 kbp. Virions are spherical with a diameter of approximately 75 nm and comprise a host-derived internal lipid membrane surrounded by a proteinaceous capsid shell. Members of the family *Turriviridae* infect extremophilic archaea of the genera *Sulfolobus* and *Saccharolobus*. Viral infection results in cell lysis for Sulfolobus turreted icosahedral virus 1 infection but other members of the family can be temperate. This is a summary of the International Committee on Taxonomy of Viruses (ICTV) Report on the family *Turriviridae*, which is available at ictv.global/report/turriviridae.

## Virion

**Fig. 1.**
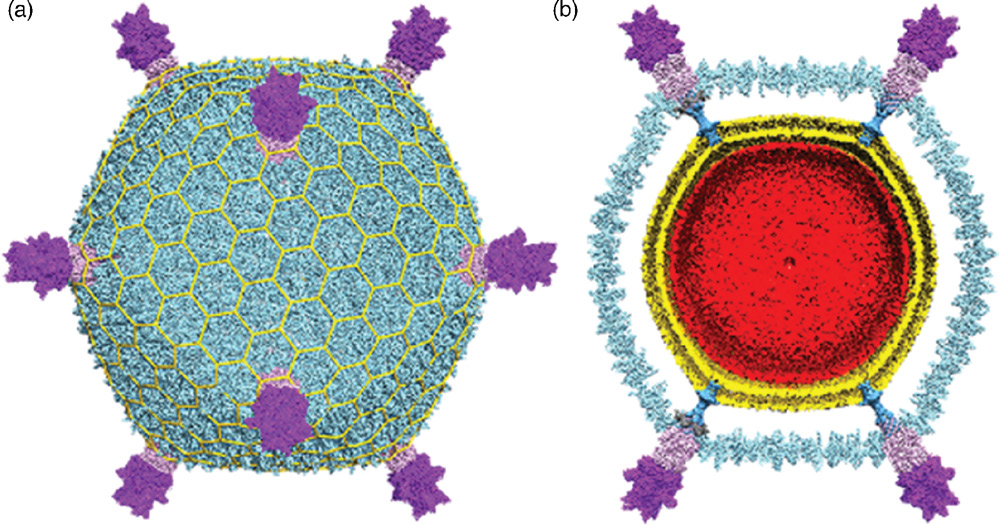
Cryo-EM reconstruction of Sulfolobus turreted icosahedral virus 1. (**a**) Virus reconstruction with turrets at five-fold points of symmetry. (**b**) Cross-section revealing the internal lipid layer (gold) and the dsDNA genome (red). Adapted from [4].

Virions of viruses in the family *Turriviridae* are morphologically similar to sphaerolipoviruses, with the main difference being the structure of the major capsid protein, which contains a double-jelly-roll fold [1]. Virions are spherical (75 nm in diameter) with turret-like protrusions extending about 12 nm above the capsid shell (Table 1, Fig. 1). The virions consist of an external proteinaceous capsid shell based on a pseudo *T*=*31d* icosahedral symmetry and an internal host-derived lipid membrane [2,3]. Encased in this spherical virion is a circular dsDNA. Sulfolobus turreted icosahedral virus 1 (STIV1) and Sulfolobus turreted icosahedral virus 2 (STIV2) are nearly identical in morphology yet behave slightly differently [4]. Virions of STIV1 were shown to bind to the pili of the host, which are likely to serve as the primary receptor [5].

**Table 1.** Characteristics of members of the family *Turriviridae*

Example	Sulfolobus turreted icosahedral virus 1 (AY569307), species *Alphaturrivirus yellowstonense*, genus *Alphaturrivirus*
Virion	Turreted icosahedral particle (about 75 nm in diameter) with an internal membrane layer
Genome	Circular, dsDNA genome of 16–17 kbp
Replication	Lytic (Sulfolobus turreted icosahedral virus 1) or temperate
Translation	Not described
Host range	Hyperthermophilic and acidophilic archaea of the genera *Saccharolobus* and *Sulfolobus*
Taxonomy	Realm *Varidnaviria*, kingdom *Bamfordvirae*, phylum *Preplasmiviricota*, class *Tectiliviricetes*, order *Belfryvirales*: the genus *Alphaturrivirus* includes the species *Alphaturrivirus yellowstonense* and *Alphaturrivirus hveragerdiense*

## Genome

**Fig. 2.**
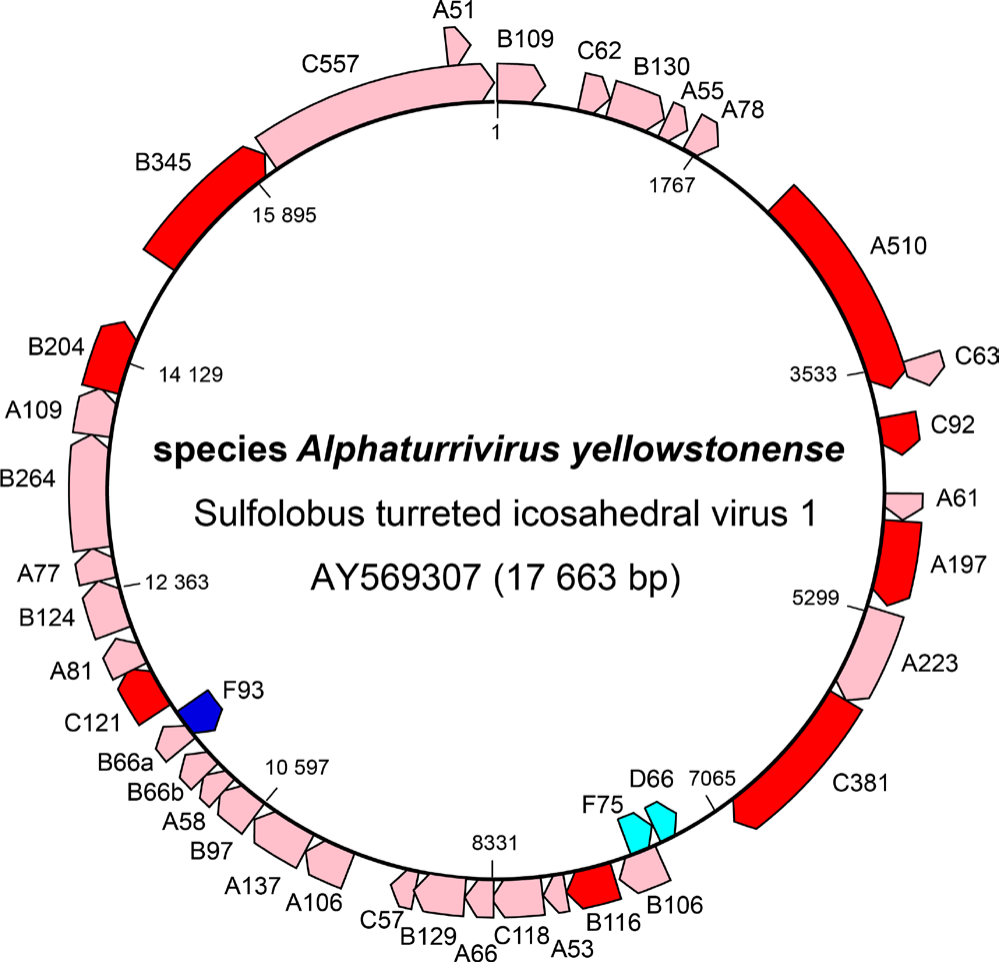
Genome organization of Sulfolobus turreted icosahedral virus 1, a member of the family *Turriviridae*. Dark red, dark blue: CDSs for which genetic or structural information is known.

STIV1 contains a circular dsDNA genome comprising 17 663 bp with 38 identified coding sequences (CDSs) (Fig. 2); STIV2 contains a circular dsDNA genome of 16 600 bp with 34 CDSs, 24 of which display homology to CDSs of STIV1, with nucleotide sequence identities above 70 % for some, including that encoding the major capsid protein [4]. Only 4 CDSs match those of other viruses.

## Replication

STIV1 displays a lytic replication cycle, resulting in seven-sided pyramid protrusions on the outer host cell surface that open like the petals of a flower to allow assembled virions to escape [6]. Pyramid structures are formed in the lipid membrane by the C92 protein and break through the crystalline S-layer present on the outer surface of the *Sulfolobus* host [7]. A putative member of the family has been shown to encode an integrase and to be integrated in the genome of *S. acidocaldarius* [8,9], indicating that turriviruses can be either virulent or temperate.

## Taxonomy

Current taxonomy: ictv.global/taxonomy. The genus *Alphaturrivirus* includes the species *Alphaturrivirus yellowstonense* and *Alphaturrivirus hveragerdiense*. Members of different species in the genus *Alphaturrivirus* can be distinguished by their host range and nucleotide sequences.

## Resources

Full ICTV Report on the family *Turriviridae*: ictv.global/report/turriviridae.

## Abbreviations

CDS: coding sequence
STIV1: Sulfolobus turreted icosahedral virus 1
STIV2: Sulfolobus turreted icosahedral virus 2
